# Sleep knowledge, attitudes and behaviours in rugby league: influences of age, body composition and ancestry

**DOI:** 10.1080/21642850.2025.2574845

**Published:** 2025-11-06

**Authors:** Alice Sharples, Rob Duffield, Jarrod Wade, Hugh H.K. Fullagar

**Affiliations:** a University of Technology Sydney, Faculty of Health, School of Sport, Exercise and Rehabilitation, Ultimo, Australia; b High Performance Department, South Sydney Football Club, Sydney, Australia; c Aspetar Orthopaedic and Sports Medicine Hospital, Doha, Qatar; d Physical Activity, Physical Education, Sport and Health (PAPESH) Centre, Department of Sport Science, Reykjavik University, Iceland

**Keywords:** Assessment, behaviour, body composition, team sport

## Abstract

**Purpose:**

This study described the relationship of age, body composition and ancestry on sleep behavior, knowledge, and attitudes in rugby league athletes.

**Methods:**

Fifty rugby league athletes completed the Sleep Practices and Attitudes Questionnaire alongside demographic information (age, body composition, ancestry). The results were compared based on age (<20 years old, 20−24 years old and >25 years), body composition (body fat percentage (%)) and ancestral groups (Pasifika, Aboriginal and/or Torres Strait Islander (ATSI) and Anglo-European). Spearman's correlation determined the associations between body composition and sleep knowledge, beliefs and attitudes. An ANCOVA compared differences between ancestral groups with age and body composition as covariates, while a one-way ANOVA was used for age group comparisons.

**Results:**

Younger athletes had higher scores for eating/drinking in bed (*p* = 0.039), while those with higher body fat percentage read less in bed (*p* = 0.022) and reported lower sleep quality (*p* = 0.027). For ancestry, significantly lower sleep difficulty scores were reported for Anglo-European compared to both ATSI and Pasifika (*p* < 0.05) athletes. Furthermore, significantly higher self-reported sleep durations existed between Anglo-Europeans and Pasifika (*p* = 0.030). Ancestry affected coping with chronic insomnia (prioritize sleep, reduce caffeine), activities in bed (eat/drink, work/thinking) and the physical environment (dark, physically comfortable, comfortable temperature).

**Conclusion:**

These findings underscore the importance of accounting for cultural and physiological variation when designing sleep education and interventions in rugby leagues. Future research needs to consider ancestry, body composition and age when assisting sleep educational programs during rugby league.

## Introduction

Sleep is essential for athlete health, performance, and recovery; however, insufficient sleep duration, low sleep quality, and frequent disturbances are common in athletic populations (Adams et al., 2017; Metse & Bowman,^2020^). In rugby league, the high physical and cognitive demands of training and competition place further importance on sleep to support recovery and performance (Fletcher et al., 2016; Glassbrook et al., 2019; Kellmann et al.,^2018^). Sleep durations in rugby league populations are typically reported below the recommended 8 h per night (Hirshkowitz et al., 2015) (6−7 h per night [Caia et al., 2018; Conlan et al.,^2022^]), which mimic findings in other team sports with similar training and competition schedules (Aloulou et al., 2021; Knufinke et al.,^2018^; Lastella et al.,^2020^). Despite the sub-optimal sleep durations reported in rugby league athletes, interventions to increase sleep duration have shown limited success, partly because they overlook individual-level factors, i.e. ancestry, age, lifestyle, and daily schedules (Blunden et al.,^2022^; Conlan et al.,^2022^; Juliff et al.,^2015^; Vlahoyiannis et al.,^2021^; Whinnery et al.,^2014^). Hence, improving sleep is often constrained by a lack of understanding of the specific demands of the population, instead of relying on the transfer of knowledge from the provider to the consumer (Blunden et al.,^2012^). Given the highly variable age, body size and ancestry in rugby league, the exploration of these factors on sleep attitudes and knowledge can aid in the design of sleep interventions to assist sustainable positive changes in sleep behaviour.

Knowledge and attitude are key theoretical drivers of health behaviours, including sleep-related activity (Ruggiero et al., 2019). In general, favourable attitudes and increased knowledge of the consequences of a behaviour are associated with positive engagement in any particular behaviour pattern (Ruggiero et al.,^2019^). In athletes, understanding knowledge and attitudes is important to understand how education interventions can be designed to better target behaviours for favourable sleep outcomes, which often exist outside of club-controlled environments. In particular, attitudes towards sleep are a predictor of sleep hygiene and the ensuing duration and quality of sleep attained in collegiate populations (Peach & Gaultney,^2017^). In the general population, sleep attitudes interact with various demographic identities, such as age, gender, ancestry, and body composition (Ruggiero et al.,^2019^). However, there is limited research investigating these demographic factors in sporting populations and, in turn, their interactions with sleep attitudes.

Ancestry is one such factor that may influence sleep patterns and behaviours. In the Australian Rugby League, Pasifika athletes comprises 46% of National Rugby League (NRL) athletes who play contracts (Lakisa et al., 2014) and 12% of indigenous athletes (‘Multicultural fact sheet,’ 2022). Research in the general population has shown a ‘sleep disparity’ in different ancestral populations, especially in the context of socioeconomic status, resulting in less quality sleep (Ruggiero et al., 2019). Current evidence suggests that inadequate sleep health (including obstructive sleep apnoea, short sleep duration and low sleep quality) may be higher in Aboriginal and/or Torres Strait Islander (ATSI) than non-ATSI Australians (Blunden et al.,^2022^). Similar findings have been reported for New Zealand Māori, who were more likely to report sleeping problems (difficulty falling asleep, waking too early in the morning, and waking refreshed) and longer lasting problems compared to non-Māori participants (Paine et al., 2005). Although these disparities have been documented in population-level studies, ancestry has received little attention in athlete-focused sleep research. With general population studies showing growing recognition that ancestry represents factors that are relevant to sleep behaviours, further investigation into the influence of ancestry on sleep behaviour in rugby league athletes is required.

Age is another important determinant of sleep health (Ovrum et al.,^2014^). Older athletes are more likely to experience lower sleep quality than younger athletes (Juliff et al.,^2015^; Simpson et al., 2010; Vlahoyiannis et al.,^2021^). Within the rugby league, younger athletes (<24 years) spent more time in bed, slept longer, and also reported higher sleep quality compared with older athletes (Conlan et al., 2022). Comparatively junior rugby league athletes went to bed later and woke later than senior rugby league athletes (>25 years), though both groups still obtained sufficient sleep duration, efficiency, and quality (Caia et al., 2017). Similar findings in sleep behaviour across general population age groups have also been reported (Ruggiero et al.,^2019^). Even within a smaller age range, rugby league athletes still face various challenges for optimal sleep behaviours (education, family, part-time training, part-time jobs, parenting, competition pressures, family, and marital status) (Conlan et al.,^2022^; Grandner,^2017^; Ruggiero et al.,^2019^). Understanding how age influences sleep-related knowledge, attitudes, and behaviours is essential for tailoring education and support across development levels.

Body composition may also play a role in sleep behaviours and outcomes due to physiological factors associated with higher body fat levels, which have been shown to negatively impact sleep quality and duration (Simpson et al., 2010). Rugby league is a collision-based sport requiring high muscular strength and power, with body mass being a key determinant of playing standards in rugby league (Caia et al.,^2020^). In general populations, those with a higher body mass index have a lower sleep efficiency, higher wake after sleep onset, higher risk of obstructive sleep apnoea, later wake times, shorter sleep duration, and longer sleep latency (Wirth et al., 2015). However, the relationship between body composition and sleep knowledge, attitudes, or behaviours still remain to be explored. Inadequate sleep behaviours have been shown to impact metabolic hormones related to appetite and food consumption both epidemiological (Taheri et al., 2004) and experimental sleep restriction (Simpson et al., 2010). Further research would help to extend our knowledge around the influence of body composition on attitudes, knowledge, and behaviors for sleep.

Despite the recognized importance of sleep and the unique challenges faced in rugby league (e.g. scheduling, training load and/or performance), there is limited research describing how sleep attitudes, behaviours, and knowledge differ across key factors such as age, ancestry, and body composition. Therefore, the primary aim of this study was to describe the sleep attitudes, behaviours and knowledge of elite rugby league athletes. A secondary aim was to examine whether sleep attitude, knowledge and behaviour vary by age group, body composition and self-identified ancestry. The primary purpose of the study was exploratory rather than hypothesis testing given the limited body of research on sleep attitudes related to demographic characteristics, sleep practices and outcomes.

## Methods

### Procedure overview

A prospective cross-sectional online survey (Sleep Practices and Attitudes Questionnaire (SPAQ)) (Grandner et al.,^2014^) was conducted to determine the sleep behaviour, attitudes, and knowledge of male rugby league athletes. The survey consisted of 15 sections and 114 questions, including (a) knowledge, (b) importance, (c) impact of sleep on performance, (d) impact on sleep, (e) self-efficacy, (f) sleep and health. Nine subscales related to sleep behaviour, (g) sleep duration, (h) sleep debt, (i) sleep quality, (j) sleepiness/tiredness, (k) coping with sleepiness, (l) coping with acute insomnia, (m) coping with chronic insomnia, (n) activities in bed, and (p) sleep environment. All the athletes completed the online survey without assistance using a personal electronic device, with data entered via Research Electronic Data Capture (REDCap).

### Participants

After a full explanation of the study, fifty-four professional rugby league athletes within one Australian professional rugby league club volunteered to participate and provided written informed consent. Parental consent was obtained for those aged <18 years of age. A power analysis was conducted using G*Power (version 3.1.9.7) to determine the required sample size for an ANCOVA that compared three ancestral groups with two covariates (age and body fat percentage). Based on a large effect size (*f* = 0.5), an alpha level of 0.05, and a power of 0.80, the analysis indicated a required total sample size of 42 participants. This ensures sufficient power to detect group differences while controlling for covariates. To estimate the required sample size for Spearman's rank analysis. Assuming a large effect size (*ρ* = 0.50), *α* = 0.05, and the desired power (1−*β*) = 0.80, the analysis suggested a minimum sample size of 23 participants. Therefore, the sample size of 50 participants met the power requirements for analysis. The criteria for inclusion involved being over the age of 16 years and currently playing for or contracting to either a National Rugby League (NRL) or a developmental team (NSW Cup, Jersey Flegg, SG Ball). The exclusion criteria included not being contracted to the club, being under 16 years old and athletes who did not complete all the required components of the study. After commencement of the study, four athletes withdrew after failure to complete the survey. The participants' age, ancestry, body fat percentage, body weight and NRL year of debut are outlined in Table 1.

**Table 1.** Participant characteristics (mean ± SD) of Australia Rugby League Athletes (*n* = 50).

	All (*n* = 50)	Age			Ancestry		
		<20years (*n* = 17)	20−24 years (*n* = 21)	>25 years (*n* = 12)	Anglo-European (*n* = 18)	ATSI (*n* = 12)	Pasifika (*n* = 20)
Age (y)	22.3 ± 4.1	18.2 ± 1.4	22.5 ± 1.6^*^	28.0 ± 2.4^*,^^†^	23.1 ± 4.4	22.8 ± 4.7	21.4 ± 3.4
Weight (kg)	98.9 ± 12.0	95.6 ± 13.5	97.7 ± 10.3	105.7 ± 10.4	99.3 ± 11.8	89.3 ± 6.8^||^	104.3 ± 11.3^‡^
Body fat (%)	16.4 ± 2.6	17.3 ± 2.5	16.2 ± 2.9	15.3 ± 1.9	15.3 ± 2.7	15.5 ± 1.8	17.8 ± 2.4^‡^,^||^
Years since NRL debut (y)	4.6 ± 4.9	0 ± 0	2.7 ± 1.9^*^	6.4 ± 2.6^*,^^†^	5.0 ± 2.7	5.5 ± 3.4	3.6 ± 2.7

* Significant difference to <20 years group (*p* < 0.05).

^†^ Significant difference to 20–24 years group (*p* < 0.05).

^‡^ Significant difference to ATSI group (*p* < 0.05).

^||^ Significant difference to Anglo-European group (*p* < 0.05).

### Ethics statement

This study was conducted in accordance with the Declaration of Helsinki and was approved by an Institutional Review Board/Ethics Committee (ETH21-6476).

### Measurements

Sleep attitudes, behaviours, and knowledge were assessed using an adapted version of the Sleep Practices and Attitudes Questionnaire (SPAQ). The SPAQ has been evaluated for concurrent and construct validity across several subscales (Grandner et al.,^2014^). Subscale 1 (sleep duration) and its components (average, weekday, and weekend sleep duration) were evaluated relative to the Pittsburgh Sleep Quality Index (PSQI) (Buysse et al.,^1989^). Subscales 6 (coping with acute insomnia), 8 (activities in bed), and 9 (sleep environment) addressed issues related to Sleep Hygiene Index (SHI) (Mastin et al.,^2006^). Subscales 12 (impact of external factors on sleep), 13 (impact of sleep on daytime functioning), and 14 (self-efficacy) captured aspects of overall sleep quality and were evaluated relative to their ability to distinguish adequate from inadequate sleepers on the PSQI. All subscales were assessed relative to the Dysfunctional Beliefs and Attitudes about Sleep scale (DBAS (Edinger & Wohlgemuth,^2001^)) to evaluate the degree to which they represent dysfunctional beliefs about sleep as measured using this questionnaire (Grandner et al.,^2014^). Several subscales were significantly correlated with the DBAS. For its internal consistency, the SPAQ had an acceptable content validity, with a Cronbach's alpha range of 0.251–0.864 for the difference subscales, with most subscales in the moderate range (Mdn = 0.629) (Ali et al., 2020). In this study, the SPAQ was adapted to the specific rugby league demographic to include questions on the effect of sleep on performance, the inclusion of rugby league staff and social media as sleep information sources and adapted to fit in with extra technology (iPad/Phone). Each subscale had its own separate score; thus, there was no presence of impact on the overall score from these customized items (Grandner et al.,^2014^). This adaptation allowed for a greater understanding of sleep attitudes, knowledge, and behaviours in the rugby league demographic in their current training environment.

### Group category classification

A secondary aim of this study was to compare sleep behaviour, attitude and knowledge based on sub-categories of age, body composition and ancestry. The following group classification definitions were used:

### Age

In NSW, Australia, the rugby league competition level is categorized based on age with U18 and U20 competitions and then by the competition levels, e.g. NRL, NSW Cup, and local club competitions (NSWRL). Age has been shown to be predisposed to low sleep quality (Juliff et al.,^2015^; Swinbourne et al.,^2016^; Vlahoyiannis et al.,^2021^). In addition, parenthood has been shown to be a contributing factor to low sleep quality (Swinbourne et al.,^2016^). To reflect the competition level, for the purpose of this study, the specific age categories were defined as <20 years old, 20−24 years old and >25 years old. As these age groupings were competition-driven, direct comparisons with the age subgroups used in prior research may be limited.

### Body composition

Body composition (i.e. body fat percentage (%BF) and body weight) was assessed using gold standard measure of dual-energy X-ray absorptiometry (DEXA). The procedures were standardized according to Australia and the New Zealand Bone Mineral Society and best practice protocol (Nana et al.,^2015^). Calibration was performed according to the manufacturer's guidelines (DMS Imaging, MEDIX DR, Sydney Australia). The scans were analysed automatically by the software, with regions confirmed by the same technician. Athletes gave permission to self-report or release their body fat percentage determined from DEXA testing undertaken by the club for internal purposes.

### Ancestry

The classification of ancestral groups in this study was informed by the current population demographics of the NRL. Ancestry was determined through self-reported data, which were based on participants' self-identification of their place of birth and ancestral background. From this information, the participants were categorized into one of three ancestral groups: 1) Pasifika, 2) Aboriginal and/or Torres Strait Islander (ATSI) and 3) Anglo-European. For the purposes of this study and its Australian context, the term ‘Pasifika’ refers to participants who identified with ancestral backgrounds from New Zealand Māori, Samoa, Tonga, Cook Islands, Fiji, Papua New Guinea, Solomon Islands and Niue (Lakisa et al., 2014). The term ATSI refers to participants who identified with the ancestral backgrounds of either Aboriginal and Torres Strait Islander peoples (Salmon et al., 2018). The term Anglo-European refers to participants who identified with ancestral backgrounds from the European Union, U.K., U.S.A., Canada, Australia, and New Zealand and do not have Aboriginal, Torres Strait Islander and/or Pasifika heritage (Lakisa et al., 2014). The classification of ancestral groups captured all major subgroups of the NRL populations; however, diverse sociocultural, environmental, and sleep-related norms could exist within these subgroups. Future research with larger and more diverse samples is warranted to explore these differences further and enhance the specificity of subgroup analyses.

### Statistical analysis

Statistical analyses were performed using the Statistical Package for the Social Sciences (SPSS, version 27, Chicago, IL, USA). All the data were assessed for normality using the Shapiro–Wilk test. The results are reported as the mean ± standard deviation (SD), with statistical significance set at *p* < 0.05. Where appropriate, 95% confidence intervals (CIs) were reported to indicate the precision of the estimates. Where appropriate, the effect size was interpreted as (small: *r* = 0.10–0.29, moderate: *r* = 0.30–0.49 and large: *r* = 0.50–1.0) (Cohen, 1988). To determine associations between body composition (body fat percentage) and sleep-related outcomes, Spearman's rank correlation coefficients (rs) were calculated for sleep knowledge, attitudes and behaviours. The coefficient of determination (*r*²) was used to report the strength of these associations. For comparisons between age groups (<20 years old, 20−24 years old and >25 years old), one-way analysis of variance (ANOVA) was conducted to assess associations with age, weight, body fat percentage, NRL debut year, sleep attitudes, and behaviours. Tukey's post hoc test was applied for multiple comparisons. If normality assumptions were violated, the Kruskal‒Wallis H-test was used with Bonferroni corrections for post hoc test comparisons. To determine differences related to ancestry groups (ATSI, Pasifika, and Anglo-European), analysis of covariance (ANCOVA) was used to assess associations between the respective groups for age, weight, body fat (%), NRL Debut (y), sleep knowledge, attitudes and behaviours. Age and body fat percentage were included as covariates based on previous research concluding an influence on sleep behaviours. Bonferroni corrections were applied for post hoc comparisons.

## Results

### Self-reported sleep duration, sleep debt and perceived sleep quality

As presented in Table 2 for age, sleep scores were not significantly different between groups (sleep duration *p* = 0.058; sleep need *p* = 0.163; sleep debt *p* = 0.837; sleep quality *p* = 0.891; sleep difficulty *p* = 0.717; sleep control *p* = 0.054). For body fat percentage, sleep quality suggested a significant moderate positive correlation with body fat percentage (*p* = 0.027, *r* = −0.314, 95% CI [2.93, 3.51]). All other body composition analyses reported no significant differences (*p* > 0.05). For ancestral groups after adjustment for age, subsequent pairwise comparisons suggested that significant differences existed in sleep difficulty (*p* = 0.001) and sleep duration (*p* = 0.031). Subsequent pairwise comparisons suggested significantly higher self-reported sleep duration for Anglo Europeans than Pasifika (*p* = 0.030). Significantly lower sleep difficulty scores were also observed for Anglo-European (95% CI [3.3, 4.2]) compared to both ATSI (*p* = 0.003, 95% CI [1.8, 3.0]) and Pasifika (*p* = 0.006, 95% CI [2.2, 3.1]). For ancestral groups, after adjustment for body composition, subsequent pairwise comparisons suggested that significant differences existed in sleep difficulty (*p* = 0.002). Subsequent pairwise comparisons suggested significantly lower sleep difficulty scores for Anglo-European (95% CI [3.2, 4.2]) compared to both ATSI (*p* = 0.003, 95% CI [1.8, 2.9]) and Pasifika (*p* = 0.025, 95% CI [2.2, 3.21]). Further ancestral analyses reported no significant differences (p ≥ 0.05).

**Table 2.** Mean ± SD of self-reported sleep duration, sleep debt and perceived sleep quality and efficacy using a 5-point Likert scale (1 = strongly agree to 5 = strongly disagree).

	All (*n* = 50)	Age			Ancestry		
		<20 years (*n = *17)	20-24 years (*n = *21)	>25 years (*n = *12)	Anglo-European (*n = *18)	ATSI (*n = *12)	Pasifika (*n = *20)
*Sleep duration and debt (h)*							
Sleep duration	7.6 ± 1.0	8.0 ± 0.5	7.5 ± 1.3	7.4 ± 0.6	8.0 ± 0.7	7.7 ± 1.2	7.3 ± 1.0^†^
Sleep need	7.9 ± 1.0	8.3 ± 0.7	7.7 ± 1.3	7.8 ± 0.8	8.2 ± 0.5	7.8 ± 1.0	7.8 ± 1.3
Sleep debt	0.3 ± 1.0	0.3 ± 0.9	0.2 ± 1.3	0.4 ± 0.7	0.2 ± 0.7	0.1 ± 1.7	0.5 ± 0.7
*Sleep quality and efficacy_(Au)_*							
Sleep quality	3.2 ± 1.0	3.1 ± 1.1	3.3 ± 1.1	3.3 ± 0.9	3.7 ± 0.9	2.8 ± 1.0	3.1 ± 1.1
Sleep difficulty	3.0 ± 1.2	2.8 ± 1.3	3.0 ± 1.1	3.2 ± 1.1	3.8 ± 0.9	2.4 ± 1.1*	2.7 ± 1.0*
Sleep control	2.2 ± 1.0	1.8 ± 0.8	2.3 ± 1.0	2.6 ± 1.1	1.9 ± 1.1	2.7 ± 0.9	2.2 ± 0.9

Abbreviation: Au, arbitrary units.

* Significant difference to Anglo-European group with both age and body composition as covariates (*p* < 0.05).

† Significant difference to Anglo-European group with only age as covariate (*p* < 0.05).

### Sleep behaviour and hygiene practices

As presented in Table 3, a Kruskal‒Wallis H-test suggested that sleep scores were not significantly different between age groups for coping with sleepiness and the physical environment (*p* ≥ 0.05). Coping with acute insomnia (do something in bed *p* = 0.045, 95% CI [2.34, 2.98]; eat/drink *p* = 0.020, 95% CI [2.39, 3.05]), coping with chronic insomnia (medications *p* = 0.045, 95% CI [3.23, 3.89]) and activities in bed (eat/drink *p* = 0.029, 95% CI [2.50, 3.26]) were significantly different between age groups. Subsequent pairwise comparisons suggested an association for acute insomnia (eat/drink *p* = 0.039, *r* = 1.16) score for participants aged <20 years old then those aged >25 years old. The activities in bed (Read) suggested a significant moderate positive correlation with body fat percentage (*p* = 0.022, *r* = 0.324, 95% CI [3.26, 4.02]). All other body composition analyses showed no significant differences (*p* ≥ 0.05). For ancestral groups after adjustment for age, significant differences existed in coping with chronic insomnia (reduced caffeine *p* = 0.028, prioritized sleep *p* = 0.026), activities in bed (Eat/Drink *p* = 0.009, comfortable temperature *p* = 0.049) and the physical environment (dark *p* = 0.025). Subsequent pairwise comparisons for age suggested that Anglo-Europeans (95% CI [1.46, 2.4]) were significantly more likely to cope with chronic insomnia through behaviour compared to ATSI (reduce caffeine *p* = 0.024, 95% CI [2.41, 3.56]). Significant differences were also suggested for behaviour between Anglo-Europeans (95% CI [1.16, 2.02]) and Pasifika (prioritizing sleep *p* = 0.024, 95% CI [2.01, 28.4]). For activities in bed (Eat/drink), pairwise comparisons for age suggested significantly less likely eat/drink behaviours for Anglo-European (95% CI [3.03, 4.14]) compared to Pasifika (*p* = 0.029, 95% CI [2.03, 3.08]) and ATSI (*p* = 0.021, 95% CI [31.69, 3.04]). For the physical environment (comfortable temperature), subsequent pairwise comparison suggested significant differences between Anglo-Europeans (95% CI [1.21, 1.96]) and Pasifika (*p* = 0.047, 95% CI [1.88, 2.59]). For physical environment (dark) pairwise comparisons suggested significant differences between Anglo-European (95% CI [1.02, 1.72] and Pasifika (*p* = 0.045, 95% CI [1.65, 2.31]).

**Table 3.** Mean ± SD of sleep practices; coping with sleepiness, acute insomnia, chronic insomnia, activities in bed, and the sleep environment using a 5-point Likert scale (1 = strongly agree to 5 = strongly disagree).

		All (*n* = 50)	Age			Ancestry		
			<20 years (*n = ***17**)	20−24 years (*n = *21)	>25 years (*n = *12)	Anglo- European (*n = *18)	ATSI (*n = *12)	Pasifika (*n = *20)
Coping with sleepiness	If I am feeling sleepy during the day:							
	I never feel sleepy	3.8 ± 1.1	3.6 ± 1.4	3.8 ± 0.9	3.9 ± 1.1	3.7 ± 1.2	3.6 ± 1.3	4.0 ± 1.0
	Sleep more or better	1.7 ± 0.8	1.5 ± 0.9	1.9 ± 0.8	1.7 ± 0.7	1.7 ± 0.8	1.9 ± 0.9	1.7 ± 0.7
	Take a nap	2.1 ± 0.9	2.2 ± 1.1	2.2 ± 0.7	1.8 ± 0.8	2.0 ± 0.8	2.2 ± 1.4	2.2 ± 0.5
	Increase caffeine	3.3 ± 1.2	3.5 ± 1.2	2.9 ± 1.1	3.7 ± 1.3	3.0 ± 1.4	3.3 ± 1.2	3.6 ± 1.1
	Increase exercise	2.6 ± 1.0	2.5 ± 1.3	2.6 ± 0.9	2.5 ± 0.8	2.6 ± 1.0	2.6 ± 1.2	2.5 ± 0.9
	If I were having trouble sleeping tonight:							
Coping with acute insomnia	Stay in bed	2.1 ± 0.8	2.0 ± 0.8	2.3 ± 1.0	1.8 ± 0.4	2.2 ± 0.8	2.2 ± 0.9	2.0 ± 0.9
	Do something in bed	2.7 ± 1.1	2.5 ± 1.2	2.4 ± 1.1	3.3 ± 0.9	2.9 ± 1.1	2.4 ± 1.2	2.55 ± 1.2
	Get up and do something	3.0 ± 1.2	2.9 ± 1.2	2.9 ± 1.3	3.6 ± 1.2	3.4 ± 1.2	2.9 ± 1.2	2.8 ± 1.2
	Eat/drink	2.7 ± 1.2	2.1 ± 1.1	3.0 ± 1.2	3.2 ± 1.1^§^	3.1 ± 1.1	2.5 ± 1.2	2.6 ± 1.2
	Drink alcohol	4.6 ± 0.6	4.6 ± 0.8	4.7 ± 0.6	4.7 ± 0.5	4.4 ± 0.8	4.5 ± 0.7	4.9 ± 0.3
	Smoke	4.8 ± 0.6	4.6 ± 0.8	4.8 ± 0.4	4.8 ± 0.4	4.6 ± 0.8	4.8 ± 0.4	4.9 ± 0.4
	Increase caffeine	4.4 ± 1.0	4.4 ± 1.1	4.3 ± 1.0	4.5 ± 0.9	4.2 ± 1.2	4.6 ± 0.5	4.4 ± 1.0
	Get up and start the day	3.2 ± 1.3	3.4 ± 1.4	3.1 ± 1.2	3.1 ± 1.3	3.1 ± 1.2	3.2 ± 1.3	3.3 ± 1.4
	If I were having trouble sleeping over a period of time:							
Coping with chronic insomnia	Medications	3.6 ± 1.1	4.1 ± 1.1	3.4 ± 1.1	3.1 ± 1.2	3.3 ± 1.2	3.6 ± 1.2	3.8 ± 1.1
	Mattress	2.8 ± 1.0	2.9 ± 1.4	2.7 ± 0.7	2.6 ± 0.9	2.5 ± 0.9	3.0 ± 1.1	2.8 ± 1.0
	Prioritise bedtime	1.8 ± 0.7	1.6 ± 0.8	2.0 ± 0.7	1.9 ± 0.8	1.6 ± 0.5	2.1 ± 0.8	2.0 ± 0.8
	Lighting	2.3 ± 1.1	2.4 ± 1.3	2.3 ± 1.0	2.3 ± 1.1	2.1 ± 0.9	2.7 ± 1.3	2.3 ± 1.1
	Temperature	2.2 ± 1.0	2.5 ± 1.3	2.1 ± 0.7	2.0 ± 1.0	1.8 ± 0.7	2.6 ± 1.1	2.4 ± 1.1
	Change sleep schedule	2.3 ± 1.0	2.1 ± 1.1	2.3 ± 0.7	2.5 ± 1.3	2.0 ± 0.8	2.5 ± 1.3	2.4 ± 1.0
	Reduce caffeine	2.4 ± 1.1	2.5 ± 1.3	2.2 ± 0.9	`2.4 ± 1.0	1.9 ± 0.9	3.0 ± 1.1*	2.4 ± 1.0
	Prioritize sleep	2.1 ± 1.0	2.1 ± 1.0	1.9 ± 0.8	2.3 ± 1.1	1.5 ± 0.6	2.1 ± 1.0	2.5 ± 1.0*
	I do the following in bed:							
Activities in bed	Read	3.6 ± 1.4	4.0 ± 1.4	3.3 ± 1.3	3.8 ± 1.4	3.6 ± 1.4	3.7 ± 1.5	3.7 ± 1.3
	Watch TV/phone/iPad/laptop	1.7 ± 0.7	1.7 ± 0.8	1.7 ± 0.5	1.8 ± 0.9	1.7 ± 0.8	1.8 ± 0.9	1.7 ± 0.6
	Eat/drink	2.9 ± 1.3	2.2 ± 1.2	3.2 ± 1.2	3.3 ± 1.4	3.7 ± 1.2	2.4 ± 1.2*	2.5 ± 1.3*
	Worry/thinking	2.9 ± 1.2	3.2 ± 1.1	2.5 ± 1.1	3.1 ± 1.4	3.3 ± 1.2	2.8 ± 1.3	2.6 ± 1.1^‡^
	Argue/angry	3.9 ± 0.7	3.9 ± 0.9	3.8 ± 0.8	4.0 ± 0.4	4.0 ± 0.8	4.1 ± 0.5	3.7 ± 0.7
	Work	3.6 ± 1.1	3.2 ± 1.4	3.7 ± 1.0	3.8 ± 0.7	4.2 ± 0.7	3.5 ± 1.0	3.1 ± 1.2
	Physical environment:							
Physical environment	Physically comfortable	1.8 ± 0.8	1.6 ± 0.6	2.1 ± 0.9	1.7 ± 0.7	1.6 ± 0.5	1.8 ± 0.8	2.1 ± 0.9^‡^
	Dark	1.8 ± 0.8	1.5 ± 0.6	2.0 ± 0.9	1.7 ± 0.8	1.4 ± 0.5	2.0 ± 1.0	2.0 ± 0.8*
	Comfortable temperature	1.9 ± 0.8	1.7 ± 0.8	2.1 ± 0.8	1.8 ± 0.7	1.6 ± 0.8	1.8 ± 0.6	2.2 ± 0.9^†^
	Quiet	2.0 ± 0.9	1.7 ± 0.8	2.2 ± 0.9	1.8 ± 0.7	1.6 ± 0.8	2.2 ± 0.8	2.2 ± 0.9

* Significant difference to Anglo-European group with both age and body composition as covariates (*p* < 0.05).

† Significant difference to Anglo-European group with only age as covariate (*p* < 0.05).

‡ Significant difference to Anglo-European group with only body composition as covariate (*p* < 0.05).

§ Significant difference to < 20years group (*p* < 0.05).

For ancestral groups after adjustment body composition, potential differences existed in coping with chronic insomnia (reduce caffeine *p* = 0.028, prioritize sleep *p* = 0.011), activities in bed (eat/drink *p* = 0.012, worry/thinking *p* = 0.040) and physical environment (Dark *p* = 0.011, physically comfortable *p* = 0.010). Subsequent pairwise comparisons for body composition suggested that Anglo-Europeans (95% CI [1.44, 2.44]) were more likely to cope with chronic insomnia through behaviours compared to ATSI (reducing caffeine *p* = 0.024, 95% CI [2.40, 3.60]). Significant differences were also suggested for behaviours between Anglo-Europeans (95% CI [1.08, 1.96]) and Pasifika (prioritizing sleep *p* = 0.009, 95% CI [2.09, 2.96]). For activities in bed (eat/drink), pairwise comparisons for body composition suggested significantly less likely eat/drink behaviours for Anglo-European (95% CI [3.03, 4.26]) compared to Pasifika (*p* = 0.038, 95% CI [1.88, 3.09]) and ATSI (*p* = 0.031, 95% CI [1.66, 3.14]). For the physical environment (Dark), pairwise comparisons suggested significant differences between Anglo-European (95% CI [0.96, 1.66]) and Pasifika (*p* = 0.016, 95% CI [1.51, 2.36]). Additionally, within the physical environment (physically comfortable), pairwise comparisons suggested significant differences between Anglo-European (CI [1.07, 1.79]) and Pasifika (*p* = 0.008, 95% CI [1.92, 2.62]), respectively. Further ancestral analyses with body composition as a covariate reported no significant differences (*p* ≥ 0.05).

### Sleep knowledge

As presented in Table 4, the Kruskal‒Wallis H-test showed that sleep scores were not significantly different between age groups (*p* ≥ 0.05). There was no significant association between nutrition knowledge scores and body composition (*p* ≥ 0.05). For ancestral groups, after adjustment for age and body composition, sleep knowledge was not significantly different between ancestral groups (*p* ≥ 0.05).

**Table 4.** Mean ± SD of sleep knowledge scores; general, rugby league performance, health, external factors and daytime functioning, using a 5-point Likert scale (1 = strongly agree to 5 = strongly disagree).

	All (*n* = 50)	Age			Ancestry		
		< 20years (*n = 17*)	20−24 years (*n = 21*)	25 + years (*n = 12*)	Anglo-Euro (*n = 18*)	ATSI (*n = 12*)	Pasifika (*n = 20*)
General sleep knowledge	2.5 ± 1.0	2.4 ± 1.1	2.6 ± 0.9	2.6 ± 1.0	2.7 ± 1.0	2.5 ± 1.0	2.4 ± 1.0
Knowledge of sleep on rugby league performance	1.6 ± 0.8	1.7 ± 0.8	1.6 ± 0.7	1.7 ± 1.0	1.4 ± 0.6	1.9 ± 1.1	1.7 ± 0.7
Knowledge of sleep on health	2.4 ± 0.8	2.4 ± 0.8	2.5 ± 0.8	2.4 ± 1.0	2.3 ± 0.7	2.5 ± 0.9	2.4 ± 0.9
Knowledge of sleep on external factors	2.9 ± 1.0	2.9 ± 1.0	2.9 ± 0.9	2.8 ± 0.9	3.0 ± 1.0	2.7 ± 1.1	2.9 ± 0.9
Knowledge of sleep on daytime functioning	2.0 ± 0.8	1.8 ± 0.8	2.2 ± 0.8	2.1 ± 1.0	1.9 ± 0.9	2.1 ± 0.9	2.1 ± 0.8

## Discussion

The present study aimed to describe the sleep attitudes, behaviour and knowledge of rugby league athletes while also comparing these factors based on age, body composition and self-identified ancestry. Our findings provide several insights into how age, body composition and ancestry can impact sleep behaviours and attitudes. First, the overall results showed that athletes self-reported ‘sleep duration’ (7.6 ± 1.0 h) and ‘hours needed’ (7.9 ± 1.0), which is within adult sleep recommendations (Hirshkowitz et al., 2015). However, ‘sleep quality’ (neither restless nor restful) and ‘sleep difficulty’ (unsure) were not highly rated among the participants. Second, younger athletes tended to eat/drink more in bed than older athletes, but this was not related to knowledge or attitude outcomes. Third, those with a higher body fat percentage reported lower sleep quality and lower rates of reading-in-bed behaviours. Finally, differences between ancestral groups were evident, based on responses to coping with chronic insomnia, activities in bed and the physical environment. These exploratory findings suggest that ancestry, body composition and age may be associated with sleep attitudes and behaviours and may need to be considered when designing sleep education programs for rugby League.

Overall, participants' attitudes towards behaviours for optimal sleep were aligned with behaviours that optimise sleep, including maintaining a regular sleep‒wake cycle; reducing stress and arousal; avoiding caffeine in the hours prior to bed; and sleeping in a cool, dark, and quiet bedroom (Mastin et al.,^2006^). The participants' attitudes toward their sleep environment and activities in bed also aligned, e.g. making the room dark, quiet, etc. (Mastin et al.,^2006^). Further, attitudes for coping with sleepiness and acute/chronic insomnia aligned with behaviours, i.e. prioritising bedtime, reducing caffeine, etc. (Mastin et al.,^2006^). However, participants' attitudes toward some behaviours did not align, i.e. watching a TV/Phone/iPad/Laptop in bed and eat/drink in bed. The present study indicated that athletes agreed that general sleep knowledge (2.5 ± 1.0), the influence of rugby league on sleep (1.6 ± 0.8), sleep health (2.4 ± 0.8), the influence of external factors (2.9 ± 1.0), and daytime functioning (2.0 ± 0.8) were associated with sleep outcomes. The difference between knowledge and poor attitudes aligns with the broader health psychology literature, which has long highlighted the ‘intention–behaviour gap’, where individuals may possess knowledge or positive intentions but fail to enact behaviour change (Feil et al.,^2023^; Mead & Irish,^2020^). An implication of these results may be the need to move beyond reliance on educational strategies only. Health behaviour theories (e.g. social cognitive theory, health belief model) emphasis that knowledge is only one component of behavioural change (Mead & Irish,^2020^). Additional components, such as personal cognitive factors, socioenvironmental factors and behavioural factors, also positively influence behavioural changes, including diet and exercise (Mead & Irish, 2020). In rugby league, prior studies have primarily implemented sleep hygiene education as a stand-alone intervention strategy (Caia et al.,^2018^; Ruggiero et al.,^2019^), with limited evidence of long-term effectiveness. Therefore, the application of health behaviour theories may provide a more robust framework for guiding interventions by addressing not only knowledge but also the cognitive, social, and environmental determinants of sustained behaviour change. With this further research, practitioners may gain a deeper insight into how attitudes and knowledge interact to influence behaviour change and identify which intervention strategies are most effective in bridging the intention–behaviour gap for promoting long-term improvements in sleep for rugby league athletes.

Ancestral groups have suggested differences in sleep attitudes and behaviours, with Anglo-European athletes reporting higher sleep difficulty scores than ATSI and Pasifika athletes and longer sleep durations and higher frequencies of worry/think in bed than Pasifika. Anglo-Europeans reported more favourable sleep hygiene practices (prioritize sleep, reduce caffeine), bed activities (work/eat/drink), and sleep environments (dark, comfortable temperature and physically comfortable) compared to their ATSI and Pasifika. Previous studies have reported differences in sleep health (OSA, quality and quantity), sleep behaviours (higher SHI scores, difficulty falling asleep, waking too early in the morning, and waking refreshed), and sleep attitudes scores across different ancestries (Blunden et al.,^2022^; Fox et al., 2018; Paine et al., 2005; Ruggiero et al.,^2019^; Whinnery et al.,^2014^); however, these have not been reported in an athletic population. The application of cultural health model frameworks may help explain ancestral-related variations in sleep behaviours. In Pasifika culture, health is a concept that considers the following factors: physical, mental and social as well as family, spiritual health and culture (University of Otago and Ministry of Health, 2012). A well-known example is the Fonofale model of health, which illustrates how sleep behaviours may be shaped not only by individual practices but also by collective obligations, spiritual beliefs, and cultural identity (Ioane & Tudor, 2017). Similarly, ATSI perspectives on health emphasize health and well-being as framed within relational and collective contexts, where family obligations and cultural norms, including spiritual, environmental, social, economic and physical factors, can take precedence over individual routines such as sleep (Swan & Raphael, 1995; Thorpe et al., 2019). The incorporation of cultural needs into sleep interventions should align with broader concepts of health, engage cultural and community leaders, and ensure that education is delivered in culturally relevant formats and contexts (e.g. family inclusive workshops, yarning circles). However, given the small sample sizes of the ancestral groups, the current findings are exploratory; therefore, future research is needed to determine cultural differences towards sleep in rugby league populations.

The present study showed that younger athletes tended to eat/drink more in bed than older athletes, despite no further differences in attitudes and/or knowledge based on age. Previous research in athletes reported that age affects the prevalence of sleep quality, with older athletes experiencing lower sleep quality than younger athletes (Juliff et al.,^2015^; Swinbourne et al.,^2016^; Vlahoyiannis et al.,^2021^). In part, this has been related to lifestyle demands, as older athletes often experience greater parenthood and family commitments, which can contribute to reduced sleep quality (Swinbourne et al.,^2016^). Current research on age and sleep in the general population reports that young adults (18−25 years) have the highest sleep duration than those aged 25−64 years (Basner et al., 2014). All athletes in this study were <35 years old and are therefore not directly comparable with ‘older adults’, which may explain the lack of overall differences in most measures. Furthermore, the age groups in this study were dictated by competition classifications and therefore may not be directly comparable to those used in prior research subgroups. Research suggests that the college students, who are similar in age to those in the current study, are the highest risk group for chronic sleep loss, lower sleep quality and most susceptible to insufficient sleep, irregular sleep schedules, and sleep problems (Peach & Gaultney,^2017^). Attitudes towards sleep in college students have emerged in current research, with studies showing that the knowledge was not predictive of sleep, attitudes yielded medium effect sizes when correlated with sleep duration, sleep quality, and sleep hygiene behaviours and students with incorrect beliefs and attitudes were more likely to have inadequate sleep (Peach & Gaultney, 2017). Young athletes may benefit from monitoring and targeted education around screen use and eating behaviours before bed. Excessive screen time before sleep is associated with increased sleep onset latency and reduced melatonin secretion (Hirshkowitz et al.,^2015^), while eating late at night has been linked to disrupted sleep patterns and reduced sleep efficiency (St-Onge et al., 2016). Implementing screen time limits, promoting evening wind-down routines, and encouraging earlier meal timing may help mitigate these risks. Knowing that the age group of athletes has the potential for a high risk of sleep problems, future work can refine the understanding of the effects of age on sleep behaviours, attitudes and knowledge in athlete populations.

Finally, body composition analysis showed higher body fat percentage athletes tended to read less in bed and also reported low sleep quality, without differences in sleep knowledge. Previous studies (Wirth et al., 2015) reported that inadequate sleep outcomes (e.g. low sleep duration) are more prevalent in those individuals with higher BMIs and sleep has the potential to impact metabolic hormones (e.g. leptin and ghrelin) related to appetite and food consumption in both epidemiological (Taheri et al., 2004) and experimental sleep restrictions (Simpson et al., 2010). These links between adiposity, appetite and knowledge/behaviour remain tenuous and require more research in both general and athletic populations. To the authors' knowledge, this is the first research to explore behaviours, attitudes and knowledge in relation to body composition in athletes. Athletes with higher body fat percentages may benefit from integrated interventions that address both sleep and nutrition. The support of sleep hygiene alongside body composition monitoring may enhance athlete health and recovery. Given the lack of explanatory research on the relationship between sleep behaviours and body composition, the current findings are speculative as to whether athletes' sleep behaviours, knowledge and attitudes differ depending on body composition.

## Limitations

Although this study is the first to investigate age, body composition and self-identified ancestry on sleep knowledge and behaviours, it is not without limitations. First, only rugby league athletes were examined from one club, which could lead to a sample bias, as body composition, ancestry and age profiles differ between sports, and the results may vary based on sub-groups within the sport. Furthermore, the use of one club limited the sample size; therefore, the results should be interpreted as exploratory, and caution is required for result interpretation particularly within the sub-groups (age, ancestry and body composition). Second, only the SPAQ was used as the validated survey; and while the strengths of the survey lie in its ability to characterize how an individual relates to sleep and sleep-related behaviour, further validated surveys could be used for specific attitudes, beliefs and knowledge of sleep behaviours. The SPAQ was adapted to include rugby league–specific items; however, the psychometric properties of these specific (albeit minor) additions have not been validated and may represent a limitation of this study. The SPAQ uses a Likert scale to determine differences in behaviours; however, when only small numerical variations are observed, interpretation of these results needs to be considered cautiously. Third, given that there is no definite threshold for body fat percentage ranking in rugby League, a median-split technique for body composition was used; though, further research into body composition is needed for future analysis. Finally, the study relies on self-reported data, which may introduce biases, e.g. recall bias. Ideally, future research would use the characteristics found in this study and trial them in an intervention conducted with a larger rugby league-wide sample to explore more detailed sub-population responses. Future research should incorporate objective sleep measurements, e.g. actigraphy, to build on these results.

## Conclusion

This study provides exploratory insights into the associations of athlete age, ancestral background and body composition with sleep knowledge, attitudes and behaviours. Overall, athletes self-reported average sleep duration of 7.6 ± 1.0 h and hours needed of 7.9 ± 1.0, aligning with adult sleep recommendations. However, perceived sleep quality (neither restful nor restless) and sleep difficulty (unsure) were not highly rated. Younger athletes reported engaging more frequently in eating/drinking in bed, although this was not associated with differences in sleep knowledge or attitudes. Ancestry emerged as a novel association, as differences in sleep behaviours as ATSI and Pasifika athletes, had higher scores for eating/drinking in bed and sleep difficulty, despite no differences in sleep knowledge when compared to Anglo-Europeans. Furthermore, body composition differences were observed in behaviours, as those with a higher body fat percentage read less in bed and also reported lower sleep quality scores. These findings underscore the importance of accounting for cultural and physiological variation when designing targeted sleep education and interventions in rugby league. Given the small sample sizes and subgroups, a logical next step is to examine sleep attitudes and behaviours on a larger population size and whether the incorporation of these factors during interventions has the potential to lead to short- or long-term improvement in sleep outcomes.

## Data Availability

Due to the participant's being elite sportsman in the public domain, supporting data are not available for this research.
